# Difference in Health Inequity between Two Population Groups due to a Social Determinant of Health

**DOI:** 10.3390/ijerph111213074

**Published:** 2014-12-16

**Authors:** Ramal Moonesinghe, Karen Bouye, Ana Penman-Aguilar

**Affiliations:** Office of Minority Health and Health Equity, Center for Disease Control and Prevention, 4770 Buford Highway, Mailstop K77, Atlanta, GA 30341, USA; E-Mails: keh2@cdc.gov (K.B.) bpv4@cdc.gov (A.P.-A.)

**Keywords:** social determinants of health, health inequity, population attributable risk

## Abstract

The World Health Organization defines social determinants of health as “complex, integrated, and overlapping social structures and economic systems” that are responsible for most health inequities. Similar to the individual-level risk factors such as behavioral and biological risk factors that influence disease, we consider social determinants of health such as the distribution of income, wealth, influence and power as risk factors for risk of disease. We operationally define health inequity in a disease within a population due to a risk factor that is unfair and avoidable as the difference between the disease outcome with and without the risk factor in the population. We derive expressions for difference in health inequity between two populations due to a risk factor that is unfair and avoidable for a given disease. The difference in heath inequity between two population groups due to a risk factor increases with increasing difference in relative risks and the difference in prevalence of the risk factor in the two populations. The difference in health inequity could be larger than the difference in health outcomes between the two populations in some situations. Compared to health disparities which are typically measured and monitored using absolute or relative disparities of health outcomes, the methods presented in this manuscript provide a different, yet complementary, picture because they parse out the contributions of unfair and avoidable risk factors.

## 1. Introduction

One of the overarching goals of the Healthy People 2020 (HP2020) Initiative in the U.S. is “achieving health equity, eliminating disparities and improving the health of all groups” [1]. In the 1990’s, Whitehead articulated the goal of health equity, which is not to eliminate all health differences, but to “reduce or eliminate those which result from factors which are considered to be both avoidable and unfair” [2]. Health equity can be defined as “social justice in health” [3]. However, there is no one standard definition for health equity in the literature [4]. For this and other reasons, measurement of health equity is a challenging prospect—one that some consider untenable. Some have proposed that health disparities are a good metric for health equity [5] since their widespread presence indicates a state of inequity. (Definitions of health disparities differ, with, for example, HP2020 defining a health disparity as “a particular type of health difference that is closely linked with social economic, and/or environmental disadvantage” [6] and others defining health disparities as “differences in health outcomes and their determinants between segments of the population, as defined by social, demographic, environmental, and geographic attributes” [7].)

Health inequities have been defined as differences in health (or its determinants) that are avoidable and unfair [4,8] and as those that benefit social groups that are already more advantaged based on factors associated with privilege (such as income, wealth, occupation, education, geography, gender, race/ethnicity, or religious affiliation) [9]. For a difference to be considered an inequity, it should be reasonable to posit that social determinants play a role in generating it [8].

Social determinants of health are the “complex, integrated, and overlapping social structures and economic systems” that are responsible for most health inequities—including social and physical environments, health services, and other structural and societal factors [10]. Concentrated poverty, educational quality, and institutional racism are specific examples of social determinants.

Social advantage is a concept that is challenging to disentangle in research. A study by Krieger *et al.* provides an example of the importance of social environments in determining health. In this study, which was based on a twin registry, twins who were raised together but experienced different socioeconomic trajectories showed different health status as adults [11]. Intergenerational transmission of social advantage and health is also important to consider. Importantly, the emerging research on epigenetics may provide a biologic link that could partially account for what has long been attributed solely to generations experiencing similar social environments [12,13].

It is important to monitor changes in distributions of social determinants of health among key population subgroups defined on the basis of demographic characteristics such as race/ethnicity, gender, and geography [12], and, in particular, to compare those with more social advantage to those with less advantage. Research that elucidates contributions of inequitable upstream social determinants to particular health outcomes is also needed. Results of such monitoring and research can indicate progress (or lack thereof) in ameliorating health disparities [12] and point to ways to intervene. Our study was designed to respond to the need for methods to evaluate differences in inequity for population subgroups [12].

For purposes of this study, we operationally define health inequity for a disease *within* a population due to a risk factor that is unfair and avoidable as the difference between the disease outcome with and without an unfair and avoidable risk factor in the population. Since health inequity, thus defined, could vary across population groups, we provide a method to estimate the difference in heath inequity *between* two population groups due to a risk factor that is unfair and avoidable.

The Blinder and Oaxaca decomposition method can be used to study the contribution of risk factors to mean outcome difference between two groups [14,15]. This method involves fitting regression models to outcomes of the two populations and partitioning the regression models into explained and unexplained components. This method only allows one to calculate the contribution of each independent variable to the explained component. Our method can be used to study how the difference in prevalence of a risk factor and the relative risk associated with the risk factor in two different populations contribute to the difference in health inequity between the two populations due to that risk factor. A similar approach has been used to estimate the difference in disease incidence between two population groups due to biological risk factors [16].

## 2. Methods

Let RF be a risk factor that contributes to a difference in incidence of disease that is considered avoidable and unfair. Let *P_i_*, *i* = 1, 2, be the incidence and I_i_ be the background risk or the risk of disease for individuals not having the risk factor, RF, in the *i*th population. The background risk includes risk of genetic and other risk factors for the diseases. We measure health inequity due to RF in the *i*th population as (*P_i_* − *I_i_*) and the difference in health inequity due to RF between the two populations as:

(1) $$\text{DHI}=\left(P_{2}-I_{2}\right)-\left(P_{1}-I_{1}\right)$$

Let *G_i_*, *i* = 1, 2, be the proportion of the population with RF for the *i*th population and *R_i_* be the relative risk associated with the RF. Let *D* denotes the disease (one or zero depending on the presence or absence of the disease) and *G* denotes the RF (one or zero depending on the presence or absence of the risk factor). Then:

(2) $$P_{i}=Pr\left[D=1|\;pop=i\right]=Pr\left[D=1|G=1,\;pop=i\right]Pr\left[G=1|\;pop=i\right]+Pr\left[D=1|G=0,\;pop=i\right]\;Pr\left[G=0|\;pop=i\right]=I_{i}\left[R_{i}Gi+\left(1-G_{i}\right)\right]$$

Note that *I_i_* = Pr[*D* = 1|*G* = 0, *pop* = *i*] and *R_i_* = Pr[*D* = 1|*G* = 1, *pop* = *i*]/Pr[*D* = 1| *G* = 0, *pop* = *i*]. The background risk for the *i*th group is then given by:

(3) $$I_{i}=\frac{P_{i}}{R_{i}G_{i}+(1-G_{i})}$$

The DHI between the two populations is then given by:

(4) $$\text{DHI}\;=\;P_{2}/\left[1\;+\;\left(1/G_{2}\left(R_{2}-1\right)\right)\right]\;-\;P_{1}/\left[1+\;\left(1/G_{1}\left(R_{1}-1\right)\right)\right]$$

For example, RF could be poverty, *G*_1_ and *G*_2_ are the proportions of the two populations in poverty, and *R*_1_ and *R*_2_ are the relative risks of disease among people in poverty in the two populations compared to the people who are not in poverty.

If *R*_1_ = *R*_2_ = *R* and *G*_1_ = *G*_2_ = *G*, then DHI = $\frac{\left(R-1\right)G}{RG+\left(1-G\right)}\left(P_{2}-P_{1}\right)$.

This shows that when relative risk and prevalence of RF are identical in both populations, the DHI is directly proportional to the difference in incidence between the two populations. It is possible to express DHI in terms of population attributable risks (PARs) of the risk factor or the proportion of diseased cases in the populations that would be avoided if the risk factor could be eliminated in the populations. If *PAR*_1_ and *PAR*_2_ are the *PAR*s of the two populations for RF, then DHI can be expressed as:

(5) $$\text{DHI}=P_{2}\left(PAR_{2}\right)-P_{1}\left(PAR_{1}\right)$$

If we assume that PARs of RF are known (or fixed) for the two populations, DHI can be estimated by DHI = ${\hat{P}}_{2}$(PAR_2_) – ${\hat{P}}_{1}$(PAR_1_) and the standard error (SE) of DHI can be calculated using the formula:

(6) $$\text{SE}(\text{DHI})=\sqrt{{\left(\text{SE}\left({\hat{P}}_{1}\right)\right)}^{2}{\left(PAR_{1}\right)}^{2}+{\left(\text{SE}\left({\hat{P}}_{2}\right)\right)}^{2}{\left(PAR_{2}\right)}^{2}}$$

where ${\hat{P}}_{2}$ and ${\hat{P}}_{1}$are the estimates of *P*_2_ and *P*_1_ respectively.

The method to calculate DHI for a risk factor with two levels (presence or absence) could be extended to a risk factor with *k*-levels. Assuming the *k*th level as the referent level to estimate the relative risk for the *i*th level of the risk factor, *R_i_* ( *i* = 1,2,…,*k*−1), and the prevalence of the *i*th level of the risk factor as *G_i_* (*i* = 1,2,…,*k*−1), the DHI is given by:

(7) $$\text{DHI}=P_{2}\left(1-\frac{1}{\sum_{i=1}^{k-1}R_{2i}G_{2i}+\left(1-\sum_{i=1}^{k-1}G_{2i}\right)}\right)-P_{1}\left(1-\frac{1}{\sum_{i=1}^{k-1}R_{1i}G_{1i}+\left(1-\sum_{i=1}^{k-1}G_{1i}\right)}\right)$$

When health outcome of interest is prevalence instead of incidence, the same formula can be applied for DHI by replacing relative risk of the risk factor with prevalence ratio of the risk factor.

## 3. Results

**Figure 1:**
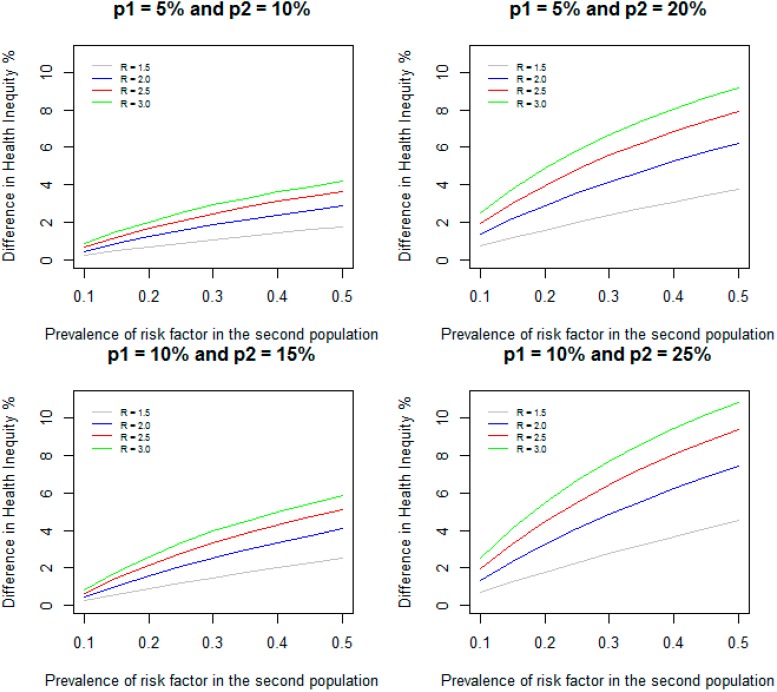
Difference in health inequity due to a risk factor between two groups when prevalence of risk factor is 10% in the first group, and varied from 10% to 50% in the second group for different values of relative risks and incidence of disease.

We assumed a prevalence of 10% for RF for the first population. For relative risks of 1.5, 2.0, 2.5, and 3.0, we varied the prevalence of RF from 10% to 50% for the second population. Figure 1 shows the DHI between the two populations when the incidence of disease for the two populations are given by 5% and 10% respectively; 5% and 20% respectively; 10% and 15% respectively; and 10% and 25% respectively. As expected, DHI increases with increasing prevalence of RF in the second population and with increasing relative risks for all the prevalence of disease considered for the two populations. For example, when relative risk is 1.5 for both populations, incidence of disease for the two populations are 5% and 10% respectively (a difference of 5% in incidence between the two populations), and the prevalence of RF is increased from 10% to 50%, the DHI due to RF increases from 0.24% to 1.76%; when relative risk is 3.0, the DHI due to RF increases from 0.84% to 4.17%. When the incidence of disease for the second population is increased to 20% (a difference of 15% in incidence between the two populations) and the prevalence of RF is increased from 10% to 50%, the DHI increases from 0.71% to 3.76% for relative risk of 1.5; for relative risk of 3.0, the DHI increases from 2.5% to 9.17%. Increases in difference in incidence between two populations lead to increases in DHI due to RF between the two populations. 

When the incidence of disease for the two populations are 10% and 15% (a difference of 5% in incidence between the two populations) and the prevalence of RF is increased from 10% to 50%, the DHI increases from 0.24% to 2.52% and from 0.84% to 5.83% respectively for relative risks of 1.5 and 3.0. Similarly, when the incidence of disease for the two populations are 10% and 25% (a difference of 15% in incidence between the two populations) and the prevalence of RF increased from 10% to 50%, the DHI increases from 0.71% to 4.52% and from 2.5% to 10.83% respectively for relative risks of 1.5 and 3.0. These results show that higher incidence of disease for the two populations leads to increased DHI between the two populations even when the differences between the incidence (5% and 15% difference in incidence) remain same. However, as indicated in the methods section, when the prevalence of RF is identical (10%) and the difference in incidence remains the same (5%) in both populations, the DHI is identical (0.24% and 0.84% respectively for relative risks of 1.5 and 3.0). 

The DHI could be larger than the difference in incidence between the two populations in some situations. The DHI is defined as: DHI = (*P*_2_ − *I*_2_) – (*P*_1_ – *I*_1_) = (*P*_2_ − *P*_1_) – (*I*_2_ – *I*_1_). We assumed that (*P*_2_ – *P*_1_) > 0, but in some situations (*I*_2_ – *I*_1_) could be negative. In these situations, DHI is greater than (*P*_2_ – *P*_1_). For example, when the incidence of disease for the two populations are 10% and 15% (a difference of 5% in incidence between the two populations), relative risk of RF is 3.0 in both populations, and the prevalence of RF is 10% in the first population, but 50% in the second population, I_1_ and I_2_ are given by 0.0833 and 0.075 respectively. Since I_2_ is less than I_1_, the DHI is 5.83% which is larger than the difference in the incidence (5%) between the two populations. 

**Figure 2:**
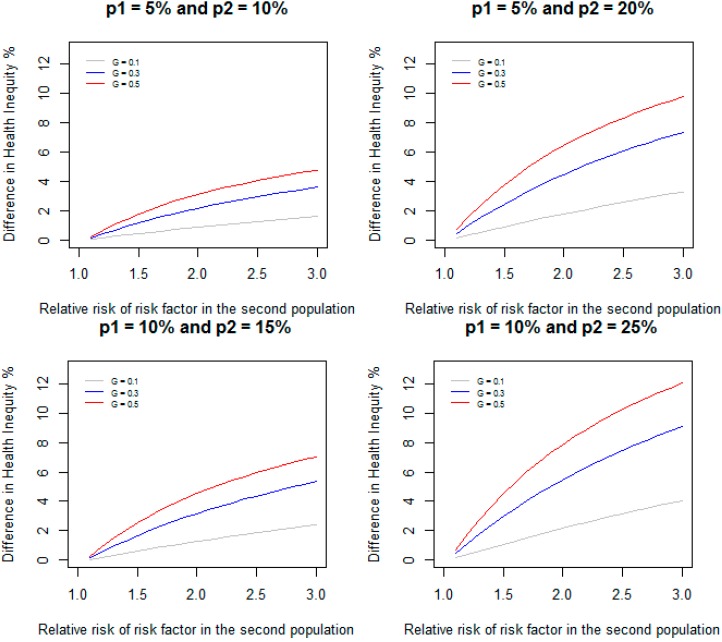
Difference in health inequity due to a risk factor between two groups when relative risk of the risk factor is 1.1 in the first group, and varied from 1.1 to 3.0 in the second group for different values of prevalence of the risk factor and incidence of disease.

Figure 2 gives DHI between the two populations when relative risk of RF in the first population is assumed to be 1.1 and is varied from 1.1 to 3.0 in the second population, and the prevalence of RF are 10%, 30%, and 50% in both populations for the same scenarios of incidence considered in Figure 1. The following example shows how this method can be applied to estimate the difference in health inequity in the prevalence of diabetes between Non-Hispanic Whites and Non-Hispanic Blacks due to poverty. 

## 4. Example

We estimated the prevalence of diabetes among adults aged 18–75 years for the Non-Hispanic black and Non-Hispanic White populations in the U.S. in 2008 using the National Health Interview Survey (NHIS) data. We categorized poverty at three levels: poor ((income is ≤ 1 × poverty level); near poor (income is 1–3 × poverty level); and not poor (income is ≥ 3 × poverty level). The prevalence of diabetes for each category and the percentage of population that falls in to each category were estimated using the NHIS data for the two populations and given in Table 1. The prevalence ratios were calculated by treating “not poor” as the referent category. The overall prevalence of diabetes for Non-Hispanic Whites and Non-Hispanic Blacks were 7.14% and 9.46% respectively. The inequity in prevalence of diabetes due to poverty in the Non-Hispanic White population is given by $0.0714\times \left(1-\frac{1}{1.5\times 0.01029+1.38\times 0.2831+0.614}\right)$ = 0.00141 and the health inequity due to poverty in the Non-Hispanic Black population is given by $0.0946\times \left(1-\frac{1}{1.3\times 0.2411+1.01\times 0.3868+0.3721}\right)$ = 0.0067. Therefore, the difference in health inequity between Non-Hispanic White and Non-Hispanic Black populations due to poverty is given by 0.0067 – 0.00141 = 0.00529, and this difference as a percentage of the difference in prevalence of diabetes (0.0232) between the two populations is 22.8%.

**Table 1:** Prevalence of diabetes, prevalence ratios, and prevalence of the risk factor among adults aged 18–75 in the U.S. in 2008.

Risk factor	Non-Hispanic White Prevalence of Diabetes: 7.14%			Non-Hispanic Black Prevalence of Diabetes: 9.46%		
	Prevalence of Diabetes (%)	Prevalence Ratio	Prevalence of Risk Factor (%)	Prevalence of Diabetes (%)	Prevalence Ratio	Prevalence of Risk Factor (%)
Poor (income is ≤1 × poverty level)	9.07	1.50	10.29	10.90	1.30	24.11
Near poor (income is 1–3 × poverty level)	8.30	1.38	28.31	8.49	1.01	38.68
Not poor (income is ≥3 × poverty level)	6.03	referent	61.40	8.39	referent	37.21

## 5. Discussion 

We provide a method to evaluate the difference in health inequity between two population groups due to a risk factor that is unfair and avoidable. Although a similar approach has been used to estimate the difference in incidence of disease between two population groups due to biological risk factors, this is the first report, to our knowledge, to apply this approach to elucidating the contribution of health inequity to a health inequality or difference. Our results show that the difference in heath inequity between two population groups due to a risk factor increases with increasing difference in relative risks and the difference in prevalences of the risk factor in the two populations. As long as the difference in incidence of disease between the two populations is not zero, the DHI due to a risk factor is not zero even when the relative risk and the prevalence of risk factor are identical in the two populations. On the other hand, it is possible to have identical incidence of disease in both populations, but non-zero DHI due to a risk factor. As shown by our results, differences in health inequity between populations due to a risk factor may present a starker picture than differences in the health inequalities themselves. This is critically important because looking only at differences in disease outcomes (without considering the contribution of factors that can be avoided) can lead to missed opportunities to advance public health and, from a social justice perspective, missed opportunities to advance health equity.

The interpretation of DHI due to a risk factor assumes that, in each population, neither the distribution of other risk factors nor their effects on disease incidence are altered by removing a risk factor. These assumptions may not necessarily always hold true. When several categorical risk factors are present, it is possible to create mutually exclusive strata by cross-classifying the categorical variables and calculate the relative risk (or prevalence ratio) in each stratum compared to the stratum having the lowest risk (or prevalence) and the corresponding prevalence of the risk factor combination [17]. Equation (7) can then be used to calculate the DHI due to multiple categorical risk factors with K strata.

The approaches used to adjust *PAR*s for other known risk factors can be extended to estimate DHI adjusted for other risk factors. Suppose we are interested in estimating DHI for a set of A risk factors, adjusting for some specified set of C risk factors because these factors could be potential confounders for the estimates of DHI for risk factors A. The health inequity due to A risk factors adjusted for C risk factors in each population then can be estimated by *P* – *P*_C,_ where *P* is the incidence of disease and *P*_C_ is the incidence that would be observed if risk factors A were eliminated [17]. The adjusted relative risks can be estimated by considering a stratum with the same levels of factors C but baseline levels of factors A [17]. This method was originally presented for case-control data but can also be used in cross–sectional studies [18]. The adjusted relative risks could also be approximated by adjusted odds ratios in a multivariate logistic regression model for rare diseases. A close variation of this method was used to estimate the proportion of deaths attributable to non-reference-weight categories (based on BMI) when other covariates are present [19,20]. These methods can be used to estimate DHI due to poverty for age adjusted prevalence of diabetes in the example given in the Results section. 

One limitation in our method of estimating DHI is our inability to estimate the standard error of DHI without assuming that PAR is known (or fixed). One could use bootstrapping to construct a confidence interval for DHI [21]. Another limitation is that these methods can only account for those unfair and avoidable risk factors that are measurable. However, these measurable factors provide a conservative estimate of the actual contribution of inequity, given that harder to measure inequities such as stigma and disenfranchisement are often related to lower socioeconomic position in the literature [22].

## 6. Conclusions

Health disparities are typically measured and monitored using absolute or relative disparities of health outcomes. The methods presented in this manuscript provide a different, yet complementary, picture of health inequalities because they parse out the contributions of unfair and avoidable risk factors (and allow for comparisons between populations of the same). Monitoring measurable social determinants of health and the DHIs due to these risk factors provides a more comprehensive way of studying health disparities between populations and it provides the opportunity to examine the extent to which particular health disparities can, at least theoretically, be avoided. A social justice perspective would suggest that this understanding is critically important for motivating action.

The current situation in the US *vis-a-vis* social determinants of health (and, by extension, social justice) supports our assertion that evaluating the contributions of health inequity to disease outcomes, as done in this study, is important for advancing health. In recent years, income inequality in the U.S. has increased. Among the top 1%, incomes grew by 31.4%, while, among the other 99%, incomes grew only by 0.4% from 2009 to 2012 [23]. Between 1980 and 2010, white families in the U.S. consistently earned approximately twice as much as black and Hispanic families. However, over time, the wealth of non-Hispanic white families has increasingly exceeded that of black and Hispanic families, and the differences are dramatic (6-fold difference by 2010) [24]. These trends in social determinants of health suggest that it is critically important to monitor DHIs between population groups to study health disparities in the United States.

## References

[1] Health People 2020. http://www.healthypeople.gov/2020/default.

[2] Whitehead M. (1992). The concepts and principles of equity and health. Int. J. Health Serv..

[3] Braveman P. (2013). What is health equity and how does a life-course approach take us further toward it. Matern. Child Health J..

[4] Braveman P. (2006). Health disparities and health equity: Concepts and measurements. Ann. Rev. Public Health.

[5] Bleich S.N., Jarlenski M.P., Bell C.N., LaVeist T.A. (2012). Health inequalities: Trends, progress, and policy. Annu. Rev. Public Health.

[6] Disparities. Health People 2020.

[7] Morbidity and Mortality Weekly Report. http://www.cdc.gov/mmwr/preview/ind2011_su.html#HealthDisparities2011.

[8] Braveman P.A., Gruskin S. (2003). Defining equity in health. J. Epidemiol. Community Health.

[9] Braveman P.A. (2003). Monitoring equity in health and health care: A conceptual framework. J. Health Popul. Nutr..

[10] Closing the Gap in a Generation: Health Equity through Action on the Social Determinants of Health. http://www.who.int/social_determinants/thecommission/finalreport/en/.

[11] Krieger N., Chen J.T., Coull B.A., Selby J.V. (2005). Lifetime socioeconomic position and twins’ health: An analysis of 308 pairs of United States women twins. PloS Med.

[12] Braveman P., Egerter S., Williams D.R. (2011). The social determinants of health: Coming of age. Ann. Rev. Public Health.

[13] Kuzawa C.W., Sweet E. (2009). Epigenetics and the embodiment of race: Developmental origins of U.S. racial disparities in cardiovascular health. Am. J. Hum. Biol..

[14] Blinder A.S. (1973). Wage discrimination: Reduced form and structural estimates. J. Hum. Resour..

[15] Oaxaca R. (1973). Male-female wage differentials in urban labor markets. Int Econ Rev..

[16] Moonesinghe R., Ioannidis J.P., Flanders W.D., Yang Q., Truman B.I., Khoury M.J. (2012). Estimating the contribution of genetic variants to difference in incidence of disease between population groups. Eur. J. Hum. Genet..

[17] Bruzzi P., Green S., Byar D., Brinton L.A., Schairer C. (1985). Estimating the population attributable risk for multiple risk factors using case-control data. Am. J. Epidemiol..

[18] Ruckinger S., Kries R.V., Tosche A.M. (2009). An illustration of and programs estimating attributable fractions in large scale surveys considering multiple risk factors. BMC Med. Res. Methodol..

[19] Flegal M.F., Graubard B.I., Williamson D.F., Gail M.H. (2005). Excess deaths associated with underweight, overweight, and obesity. JAMA.

[20] Sttenland K., Armstong B. (2006). An overview of methods for calculating the burden of disease due to specific risk factors. Epidemiology.

[21] Greenland S. (2004). Interval estimation by simulation as an alternative to and extension of confidence intervals. Int. J. Epidemiol..

[22] Smith L. (2005). Psychotherapy, classism, and the poor: Conspicuous by their absence. Am. Psychol..

[23] Striking it Richer: The Evolution of Top Incomes in the United States. http://eml.berkeley.edu/~saez/saez-UStopincomes-2012.pdf.

[24] Less than Equal: Racial Disparities in Wealth Accumulation. http://www.urban.org/publications/412802.html.

